# High Dose Zinc Supplementation Induces Hippocampal Zinc Deficiency and Memory Impairment with Inhibition of BDNF Signaling

**DOI:** 10.1371/journal.pone.0055384

**Published:** 2013-01-31

**Authors:** Yang Yang, Xiao-Peng Jing, Shou-Peng Zhang, Run-Xia Gu, Fang-Xu Tang, Xiu-Lian Wang, Yan Xiong, Mei Qiu, Xu-Ying Sun, Dan Ke, Jian-Zhi Wang, Rong Liu

**Affiliations:** Key Laboratory of Neurological Disease, Ministry of Education, China, and Department of Pathophysiology, Tongji Medical College, Huazhong University of Science and Technology, Wuhan, China; Cleveland Clnic Foundation, United States of America

## Abstract

Zinc ions highly concentrate in hippocampus and play a key role in modulating spatial learning and memory. At a time when dietary fortification and supplementation of zinc have increased the zinc consuming level especially in the youth, the toxicity of zinc overdose on brain function was underestimated. In the present study, weaning ICR mice were given water supplemented with 15 ppm Zn (low dose), 60 ppm Zn (high dose) or normal lab water for 3 months, the behavior and brain zinc homeostasis were tested. Mice fed high dose of zinc showed hippocampus-dependent memory impairment. Unexpectedly, zinc deficiency, but not zinc overload was observed in hippocampus, especially in the mossy fiber-CA3 pyramid synapse. The expression levels of learning and memory related receptors and synaptic proteins such as NMDA-NR2A, NR2B, AMPA-GluR1, PSD-93 and PSD-95 were significantly decreased in hippocampus, with significant loss of dendritic spines. In keeping with these findings, high dose intake of zinc resulted in decreased hippocampal BDNF level and TrkB neurotrophic signaling. At last, increasing the brain zinc level directly by brain zinc injection induced BDNF expression, which was reversed by zinc chelating *in vivo*. These results indicate that zinc plays an important role in hippocampus-dependent learning and memory and BDNF expression, high dose supplementation of zinc induces specific zinc deficiency in hippocampus, which further impair learning and memory due to decreased availability of synaptic zinc and BDNF deficit.

## Introduction

Zinc is one of the most important trace elements in the human body which is required for many physiological processes such as cell proliferation and differentiation, nuclear acid metabolism, growth and development, and enzymatic activity regulation [1]. Zinc deficiency, which may induce growth retardation, hypogonadism, immune dysfunction and cognitive impairment [2], has attracted extensive public attention and scientific research. To prevent the occurrence of zinc deficit, zinc fortification and supplementation is widely used, especially in the youth in developed countries [3]. Zinc supplement is also prevalent in the treatment of diarrhea and pneumonia in the children [4], [5], [6]. Thus, in condition when mean dietary zinc intake derived from food alone is higher than the minimum recommendation [7], [8], young zinc supplement users may be at a risk for excessive intake of zinc. In supporting this, zinc overuse was reported in the preschoolers [8].However, zinc supplementation was thought to be safe by most parents and caregivers, and the potential harm of zinc overdose was largely underestimated.

Zinc ions are abundant in the brain. Most of that zinc is tightly bound to proteins where zinc acts either as a component of the catalytic site of enzymes or in a structural capacity. About 10% of total brain zinc is localized to synaptic vesicles, which may be released on excitation and plays a role in modulation of synaptic signaling [9]. This pool of zinc is the only form of zinc readily stained histochemically (the chelatable zinc). The highest concentration of chelatable zinc was observed in hippocampal mossy fiber-CA3 region, where synaptic zinc releases together with glutamate and may serve to modulate responses of NMDA receptors [10], [11]. Zinc release is essential for hippocampus-dependent learning and memory. Synaptic zinc is required for the induction of mossy fiber-CA3 long-term potentiation (LTP) [12], [13]. Decreased zinc level in hippocampus correlates positively with spatial memory deficit in aged rats [14], and in situ chelating of button zinc disrupts performance of the animal in spatial memory test [15]. There is a large body of evidence showing that dietary zinc deficiency may induce learning and memory impairment [16], [17], [18], but little is known about the effects of zinc overdose on brain zinc homeostasis and memory. Two studies have reported that zinc supplemented rats showed spatial reference memory deficit [19], [20], however, the detailed mechanisms underlying the memory impairment need further exploration.

In the present study, we enhanced the zinc intake in weaning ICR mice by increasing zinc levels in drinking water for 3 months, and found unexpectedly a zinc deficiency in the hippocampus, which was accompanied with hippocampus dependent memory deficit and decreased expression levels of learning and memory related receptors/synaptic proteins. Furthermore, the zinc-regulated BDNF neurotrophic signaling was also impaired in hippocampus. Thus, hippocampus is susceptible to zinc enhancement, high dose supplementation of zinc may impair hippocampus dependent memory through decreasing synaptic zinc release and BDNF deficit.

## Materials and Methods

### Ethics Statement

All animal experiments were carried out according to the “Policies on the Use of Animals and Humans in Neuroscience Research” revised and approved by the Society for Neuroscience of China in 1995, and protocols were approved by the Institutional Animal Care and Use Committee in Tongji Medical College, Huazhong University of Science and Technology.

### Antibodies and Reagents

Rabbit polyclonal antibodies (pAb) against NMDA-NR2A, NR2B, PSD-93, PSD-95 (1∶1000), BDNF (1∶100), and mouse monoclonal antibody (mAb) against DM1A (1∶2000) were all from Abcam (Cambridge, UK). Polyclonal antibodies against AMPA-GluR1 (1∶500) and phosphorylated CREB at Ser133 (1∶1000), and mAb against AMPA-GluR2 (1∶1000) were from Millipore (Billerica, MA, USA). PAb against total CREB was purchased from Cell Signaling Technology (Danvers,MA, USA). Polyclonal antibodies against total and phosphorylated TrkB (1∶1000) were from BioVision (Milpitas, CA, USA) and Epitomics (Burlingame, CA, USA) separately. Zinc sulfate was from Sigma (St. Louis, MO, USA). Clioquinol (CQ, chelator of zinc) was purchased from Merck (KGaA, Darmstadt, Germany). N-(6-methoxy-8-quinolyl)-para-toluenesulfonamide (TSQ) was from Invitrogen (Molecular Probes, Eugene, OR, USA).

### Animals and Treatment

Weaning ICR mice (male, 21 d) were supplied by Slack King Experimental Animal Co., Ltd (Changsha, China). All animals were kept at 22±2°C on daily 12 h light-dark cycles with ad libitum access to food and water. The mice were divided into three groups (n = 16 in each group): 1. Control group, mice were given a standard diet (30 ppm zinc) and deionized water; 2. Low dose zinc supplemented group, mice were given a standard diet and deionized water containing ZnSO_4_.7H_2_O 66.2 mg/L (Zn 15 ppm); 3. High dose zinc supplemented group, mice were given a standard diet and deionized water containing ZnSO_4_.7H_2_O 265 mg/L (Zn 60 ppm). All the mice were fed for 3 months. At the end of treatment, the animal behaviors were tested.

For the brain zinc injection, SD rats (male, weight 250–300 g, 4 months old, supplied by Experimental Animal Center of Tongji Medical College) were deeply anesthetized and divided into experimental group for hippocampal injection with zinc sulfate (1 mM, 3 µl) and control group for injection with 0.9% NaCl. The experimental group was further divided into two groups with or without treatment of zinc chelator CQ (Intraperitoneal injection, 50 mg/kg/48 h). Six days after the injection, the rats were sacrificed and the hippocampus was homogenized for Western blotting.

### Behavior Tests

#### Open field test

The test apparatus was an open rectangular box (60 cm wide×60 cm long×40 cm high) with the floor divided into 100 identical squares measuring 6 cm×6 cm [21]. Before the recording, each mouse was placed individually in the centre of the field for 5 min to adapt to the environment, then the locomotor activity was video recorded. Mice were analyzed for a total period of uninterrupted 10 min. All testing was conducted between 07:00 a.m. and 7:00 p.m. The central field was defined as 24 cm×24 cm in the middle of the open field, whereas the border field was defined as 12 cm width area from the outer periphery of the open field. The analysis was performed manually by examining the video recording. Moving time and time in central field were counted with a stopwatch. Distance traveled was measured by counting the number of squares traveled.

#### T-maze test

T-maze apparatus allows to measure spatial working memory, the procedure is the same as previously described [22], [23]. The T-maze consists of a long arm 63.5 cm×10 cm, and two short arms (left and right) 55 cm×10 cm. One week before training, animals were deprived of food until their body weight was reduced to 80% to 85% of the initial level. Then, mice were habituated to the maze with three trials in which they were accustomed to reward food (small sugar pellet). Mice were tested on 4 trials per day, each trial consisting of two runs, a forced run and a choice run. For the force-run, the mouse was forced to enter either left or right arm to get the food (a small sugar pellet) by blocking a door. The direction of the forced run was random but no more than 2 times allowed in the same direction consecutively. For the choice-run, the blocked door was removed and the mouse was allowed to choose either arm freely. When the mouse entered the previously unvisited arm, the reward was given. The interval between the force-run and the choice-run was 15 s. Between each run, the arms were cleaned with 75% alcohol to remove the effect of olfactory quickly. Each block consisted of a total of eight trials, conducted in two consecutive days with four trials per day. Accuracy of response was scored and recorded by two experimenters.

#### Contextual fear conditioning

The procedure was similar to those previously used [24]. Mice were placed into a square chamber with grid floor. On the first day (day1), each mouse was habituated to the chamber for 2 min, followed by a 0.75-mA, 2-s foot shock. Then the mice were returned to their home cages. The same procedure was repeated for two times with an interval of 2 min. On the next day (day2), the mice were exposed to the chamber without any stimulus for 300s. The contextual conditioning was assessed by recording freezing behavior during the 300s exposure. Long term memory freezing scores were recorded seven days later (day9).

#### Contextual discrimination

For contextual discrimination task [25], [26], two distinct chambers (A and B) that shared features were used to test the ability of the animals to discriminate the contexts. The chambers were located in the same room and had identical grid floors, but they were different in shape (square versus triangle) and scent (70% ethanol versus 1% acetic acid). Mice first received three days of conditioning in chamber A. Conditioning took place 192 s after the mouse was placed in chamber A with a 0.75-mA, 2-s foot shock. Mice were returned to their home cages 1 min later. Across the subsequent two consecutive days (day4 and day5) mice were placed in counterbalanced order in A and B chambers. Each test consisted of a 6-min exposure to the chamber without a foot shock. On days 6 through 12, mice were placed in counterbalanced order in A and B chambers. However, they received a single foot shock during exposure to context A and never received foot shocks during exposure to context B. The order of the chamber exposed was as the following: BA-AB-AB-BA-AB-AB-BA. At least 4 h interval was required between each exposure. Freezing scores were calculated for the first 192 s during each exposure. Discrimination ratios were calculated as follows: Ratio = freezing percentage in chamber A/(freezing percentage in chamber A+B). Long term memory freezing scores were recorded seven days later (day19). Behavioral performance was analyzed by one-way ANOVA and Student’s t-test.

### Plasma and Brain Zinc Analysis

Zinc concentrations in the blood and brain were detected by atomic absorption spectrophotometry. At the end of behavior tests, mice were decapitated when they were deeply anesthetized with intraperitoneal injection of chloral hydrate (0.6 ml/kg), the brains were rapidly removed. Hippocampus and cortexes were separated. The blood was centrifuged at 12,000 g for 15 min, then the serum and hemocytes were separated. Plasma, hemocytes and brain tissues were digested at room temperature for 5 days with 0.1 mol/L or concentrated ultra-pure nitric acid, respectively. Zinc was analyzed by using flame atomic absorption spectrophotometer AA-240FS from Varian (Palo Alto, California, USA).

### Timm Staining

The mice were deeply anesthetized and then fixed by transcardial perfusion with 0.9% NaCl followed by 0.3% Na_2_S in 100 mM phosphate buffer (PB) and 4% paraformaldehyde in 100 mM phosphate buffer (PB). After perfusion, the brains were postfixed in 4% paraformaldehyde overnight at 4°C. Coronal sections of the brain were cut (30 µm thick) using Vibratome (Leica, S100, TPI). Sections were immersed in fresh Timm staining solution (50% Gum Arabic 60 ml, 5.6% 1,4- Hydroquinone 10 ml, Citrate buffer 10 ml,17% Silver nitrate 1.5 ml) for 45 min in darkness at 26°C. Then sections were washed in de-ionized water for 10 min in dark cupboard. Sections were dehydrated using a graded series of ethanol solutions, transparented using xylene, placed under cover slips and analyzed with a microscope (Nikon, 90i, Tokyo, Japan).

### TSQ Staining

Vesicular chelatable zinc was imaged using the N-(6-methoxy-8-quinolyl)-para- toluenesulfonamide (TSQ) staining method [27]. The fresh frozen mice brains were sectioned coronally. Five evenly spaced sections were collected through the hippocampal region of each brain and immersed in 4.5 µM TSQ in 140 mM sodium barbital/140 mM sodium acetate buffer (pH: 10–10.5) for 60 s, then rinsed in 0.9% saline for 60 s. The TSQ–zinc binding was imaged and photographed using a fluorescence microscope (LSM 710 META, Zeiss, Germany) with 360 nm of ultraviolet light.

### Golgi Staining

The mice were deeply anesthetized and then fixed by transcardial perfusion with 0.5% NaNO_2_ followed by 4% formaldehyde and potassium dichromate with chloral hydrate which were mixed in 4% formaldehyde. After perfusion, the brains were postfixed in potassium dichromate with chloral hydrate mixed liquid for 3 days. Then the brains were moved into 1% AgNO_3_ solution for 3 days. Coronal sections of the brain were cut (30 µm thick) using Vibratome (Leica, S100, TPI). Sections were dehydrated using a graded series of ethanol solutions, transparented using xylene, placed under cover slips and analyzed with a microscope (Nikon, 90i, Tokyo, Japan).

### Western Blotting

The hippocampus of the mice or the rat was homogenized in 10 volumes (ml/g wet tissue) homogenate buffer containing 50 mM Tris-HCl, pH 7.0, 0.5 mM PMSF, 2.5 mM EDTA, 2.5 mM EGTA, 2.0 mM Na_3_VO_4_,100 mM NaF and 1∶1000 protease inhibitor cocktail (Sigma-Aldrich, St. Louis, MO, USA). The brain extracts were mixed with sample buffer containing 50 mM Tris-HCl (pH 7.6), 2% SDS,10% glycerol,10 mM dithiothreitol and 0.2% bromophenol blue and boiled for 5 min. Boiled samples were electrophoresized in 10% SDS-polyacrylamide gel and the separated proteins transferred to nitrocellulose membranes. The membranes were then incubated with primary antibodies that were detected using anti-rabbit or anti-mouse IgG conjugated to IRDyeTM (800CW; Licor Biosciences, Lincoln, NE,USA) for 1 hour at room temperature and visualized using the Odyssey Infrared Imaging System (LicorBiosciences, Lincoln, NE, USA). The protein bands were quantitatively analyzed by Kodak Digital Science 1D software (Eastman Kodak Company, New Haven, CT, USA). The levels of the phosphorylated proteins were normalized by the corresponding total protein levels.

### Statistical Analysis

Data are expressed as mean ± SEM, and analyzed using SPSS 16.0 statistical software (SPSS Inc., Chicago, IL, USA). The one-way analysis of variance (ANOVA) procedure followed by LSD’s post hoc tests was used to determine the differences among groups. The level of significance for all analysis was set at *p*<0.05.

## Results

### High Dose Zinc Supplementation Induces Hippocampus-dependent Memory Impairment

**Figure 1:**
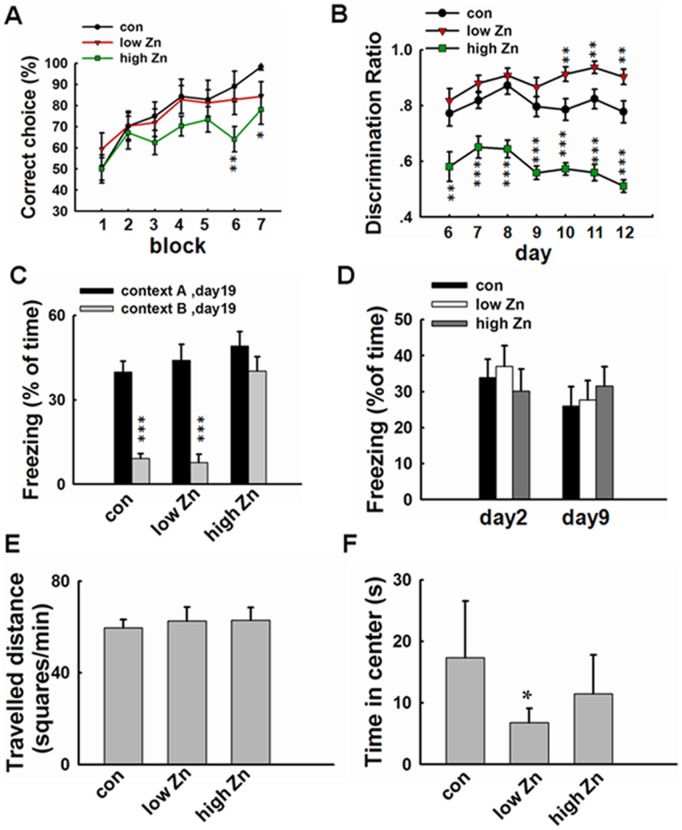
High dose zinc supplementation induces hippocampus-dependent memory impairment. (A) T-maze task for working memory assessment of zinc supplemented mice (n = 8). Compared with control mice, high-dose zinc supplemented mice exhibited working memory deficit. (B–C) Contextual discrimination test of the mice (n = 16).High-dose zinc supplemented mice showed significantly lower discrimination ratio than the other two groups across the seven days of acquisition. Low-dose zinc supplemented mice showed better discrimination than control in the last three testing days (B). Seven days later, the discrimination was recorded again, control and low-dose zinc supplemented mice could discriminate the two contexts whereas high-dose zinc supplemented mice still could not (C). (D) Contextual fear conditioning test of the mice (n = 16). There was no difference in freezing ratio among the three groups. (E–F) Open field test of the mice (n = 16). There was no difference in locomotor habituation among the three groups (E); Low-dose zinc supplemented mice showed shorter time in the center square than control, indicating increased anxiety in this group. *, *p*<0.05; **, *p*<0.01;***, *p*<0.001 vs. control group.

To evaluate the effect of zinc supplementation on the behavior of the mice, we performed a lot of behavior tests on the animals. The results showed that high dose supplementation of zinc induced hippocampus-dependent memory impairment. Spatial working memory and contextual discrimination are particularly sensitive to hippocampal function [28], [29]. Compared with control and low-dose zinc supplemented mice, mice fed with high dose of zinc showed delayed learning in the rewarded-alternation T-maze task for working memory (Fig. 1A). In the contextual discrimination test, the high-dose zinc supplemented mice showed a significantly decreased discrimination ratio during all the 7 days. On the contrary, low-dose zinc supplemented mice showed better discrimination between the two similar chambers during the last 3 days (Fig. 1B). One week after the contextual discrimination training, the long-term memory was recorded and the same result was observed. High-dose zinc supplemented mice still could not discriminate the two contexts (Fig. 1C). In the test of contextual fear conditioning, which is mediated by both hippocampus and nonhippocampus neural systems [29], no difference among the three groups was observed (Fig. 1D). When the high-dose zinc supplemented mice were placed in an open field, they showed indistinguishable center time and locomotor habituation compared with control (Fig. 1E,F), indicating that the spatial working memory deficit is not caused by altered motivation or ability to learn explicit information. Low-dose zinc supplemented mice showed decreased center time compared with control, indicating a higher anxiety level in this group (Fig. 1F). In a summary, these behavior testing results suggest that high dose supplementation of zinc induces hippocampus-dependent memory impairment.

### High Dose Zinc Supplementation Induces Zinc Deficiency in Hippocampus

**Figure 2:**
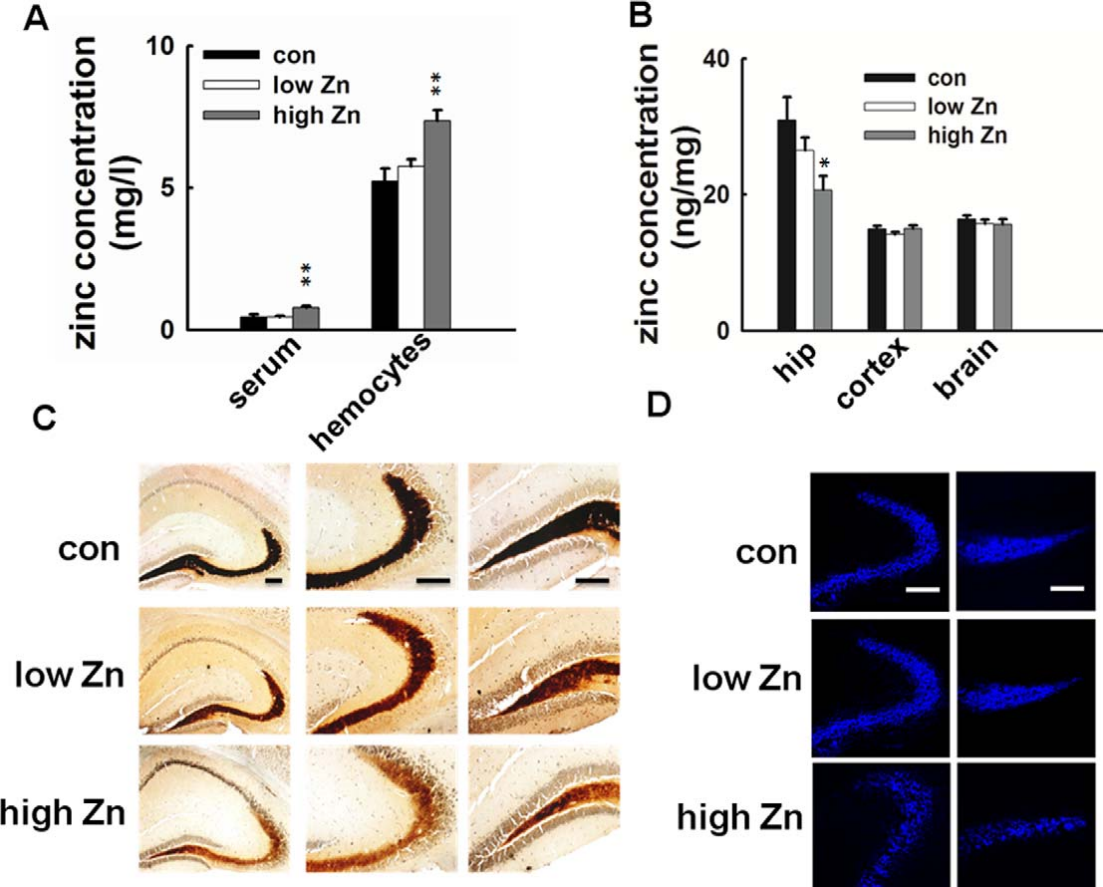
High dose zinc supplementation induces zinc deficiency in hippocampus. (A) Zinc concentrations in the serum and hemocytes were detected by atomic absorption spectrophotometry (n = 10). High-dose zinc supplemented mice showed significantly increased zinc levels both in serum and hemocytes, while low-dose zinc supplemented mice only showed a trend of zinc increase in hemocytes. (B) Zinc concentrations in the brain (n = 6). High-dose zinc supplemented mice showed decreased zinc levels in hippocampus than control mice, with no change of zinc levels in cortex and the whole brain. Low-dose zinc supplementation did not induce any change of the zinc levels in the detected brain regions. All values are mean ± SEM, *p<0.05;** *p*<0.01 vs. control group. (C)Timm Staining of the brain slices from zinc supplemented and control mice. High-dose zinc supplemented mice showed reduced zinc levels in hippocampus, especially in CA3 and DG area. Scale bar = 200 µm. (D) TSQ Staining of the brain slices. High-dose zinc supplemented mice showed dramatic loss of chelatable zinc staining both in CA3 and DG area. Scale bar = 200 µm.

To explore the mechanisms underlying the hippocampus-dependent memory deficit, we detected the zinc levels in different compartments. First, we used atomic absorption spectrophotometry to evaluate the total zinc levels in different compartments of the mice. Zinc supplementation in high dose resulted in significant increase of zinc levels both in serum and hemocytes, no change of blood zinc level was observed in low dose zinc supplemented mice, even though there was a trend of elevation in hemocytes (Fig. 2A). We observed with surprise that zinc overdose induced a specific decrease of zinc content in hippocampus with no change in the cortex or the entire brain (Fig. 2B). To confirm the hippocampal zinc deficiency in high level zinc intake mice, two classic imaging methods which detect the chelatable zinc (synaptic releasable zinc), Timm staining and TSQ staining were performed. The results showed that the chelatable zinc mainly located in CA3 and dentate gyrus (DG) in hippocampus, oral zinc overdose induced obvious deficit of synaptic releasable zinc in hippocampus, especially in CA3 and DG (Fig. 2C,2D). Thus, hippocampal zinc homeostasis is sensitive to peripheral zinc enhancement, and high dose supplementation of zinc may induce hippocampal zinc deficiency and disturb synaptic zinc release.

### High Dose Zinc Supplementation Results in Decreased Synaptic Receptors and Scaffolding Protein Levels, and Loss of Dendritic Spines in Hippocampus

**Figure 3:**
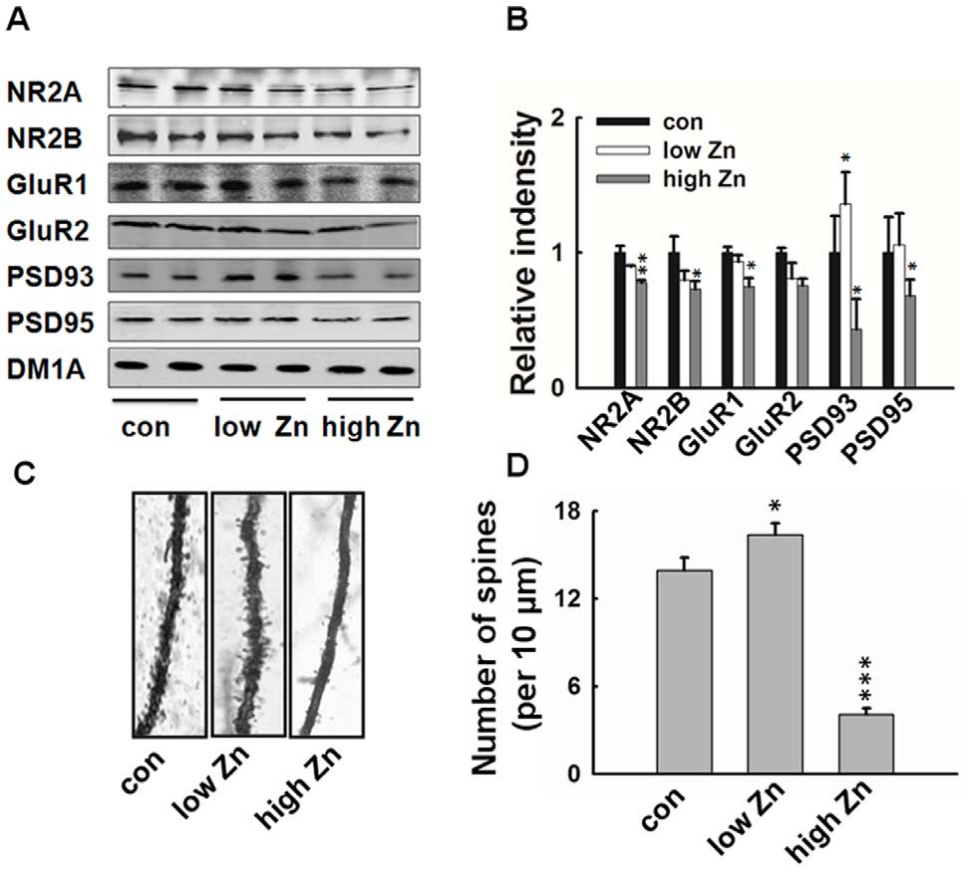
High dose zinc supplementation induces decreased levels of synaptic receptors and scaffolding proteins and retarded spine formation in hippocampus. (A–B) Western blotting (A) and the quantitative analysis (B) showed reduced levels of NMDA-NR2A, NR2B, AMPA-GluR1, PSD93 and PSD95 in high-dose zinc supplemented mice. In contrast, expression levels of PSD93 was increased in low-dose zinc supplemented mice (n = 4). (C) The representative images for morphology of dendritic spine in hippocampal region of zinc supplemented and control mice visualized by Golgi staining. (D) Quantitive analysis of the spine density (calculated as the average number of spines per 10 µm on the dendrites) in hippocampal DG area. *, *p*<0.05; **, *p*<0.01;***, *p*<0.001 vs. control group.

To further disclose the mechanisms of hippocampus-dependent memory impairment, we detected the protein levels of memory-related glutamate receptors and scaffolding proteins. As shown in Fig. 3A and 3B, high dose zinc supplementation dramatically reduced protein levels of NMDA-NR2A, NR2B, AMPA-GluR1, PSD-93 and PSD-95 in hippocampus, AMPA-GluR2 also showed an un-significant decrease. Zinc supplementation in low dosage increased the expression of PSD-93, with no effect on the other tested protein levels. Although the effect of zinc enhancement in low dosage on the memory-related synaptic proteins was not consistent, the overall decrease of these proteins in high dose zinc supplemented mice indicated that loss of the central molecular devices also contributed to the memory deficit. To further confirm this speculation, we observed the dendritic spines by Golgi staining in hippocampal DG area. The result showed that high-dose zinc supplementation induced significant loss of the dendritic spines (Fig. 3C, 3D), which was consistent with the decrease level of synaptic receptors and scaffolding proteins. These data suggest that high level zinc supplementation may disturb the formation of hippocampal dendritic spines by decreasing the expression of dendritic proteins.

### High Dose Zinc Supplementation Inhibits BDNF-TrkB Neurotrophic Signaling in Hippocampus

**Figure 4:**
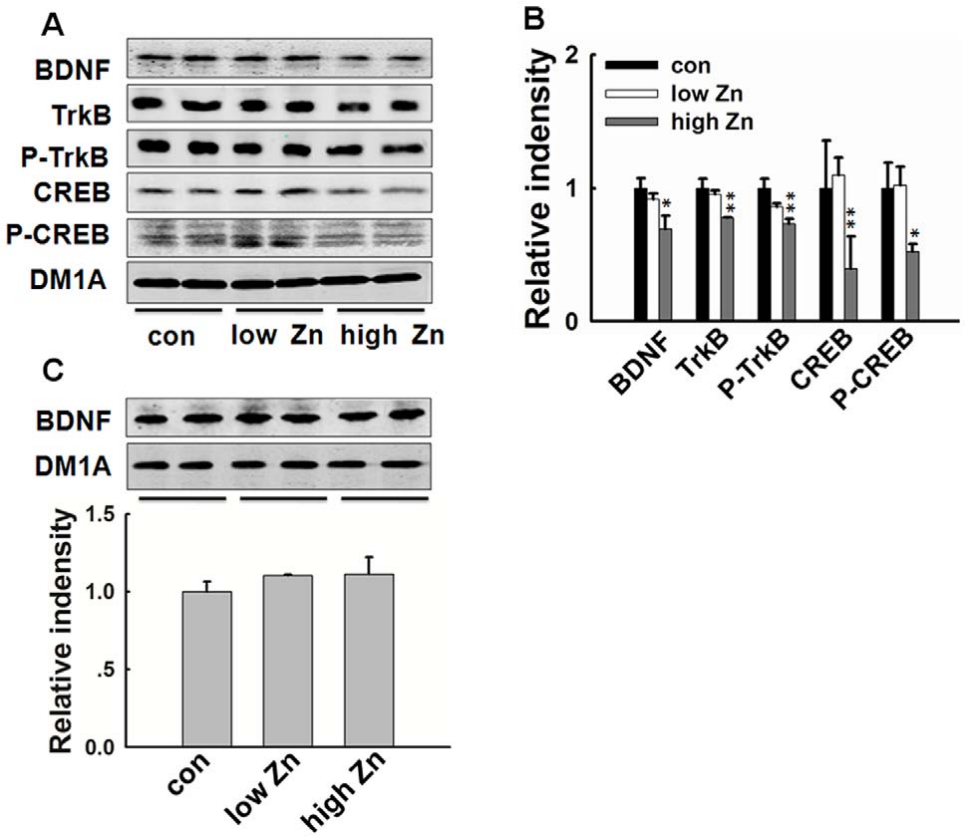
High dose zinc supplementation induces BDNF-TrkB neurotrophic signaling impairment in hippocampus. (A–B) Western blotting (A) and the quantitative analysis (B) showed reduced levels of BDNF, TrkB, p-TrkB, CREB, and p-CREB in high-dose zinc supplemented mice (n = 4). (C) Western blotting (upper) and the quantitative analysis (lower) showed no change of the BDNF level in cortex of the mice (n = 4). *, *p*<0.05; **, *p*<0.01 vs. control group.

To verify the upstream factors that may underlie the decreased expression of synaptic receptors and scaffolding proteins, we tested the BDNF-TrkB neurotrophic signaling pathway in the hippocampus of the mice. High dose zinc supplementation induced significant decrease of hippocampal BDNF, TrkB and phosphorylated TrkB levels. At the same time, the downstream CREB and phosphorylated CREB levels were also reduced. Zinc enhancement in low dosage did not interrupt the BDNF-TrkB signaling pathway (Fig. 4A, 4B). In the cortex where zinc level showed no change, high dose zinc supplementation had no effect on BDNF expression (Fig. 4C). These results suggested that BDNF-TrkB neurotrophic signaling inhibition in hippocampus by high dose zinc supplementation may be induced by zinc deficiency.

### BDNF Expression is Regulated by Zinc Directly in Rat Brain

**Figure 5:**
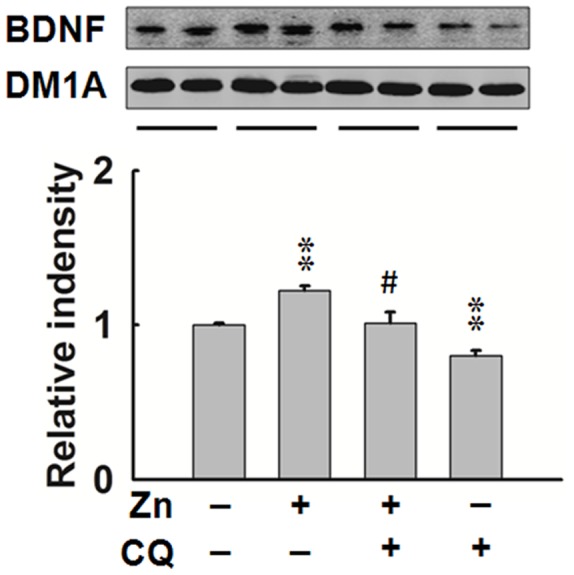
Zinc is required for the expression of BDNF in hippocampus. Upper panel, SD rats were injected with zinc sulfate (1 mM, 3 µl) or 0.9% NaCl (3 µl) into the hippocampus, with or without intraperitoneal administration of zinc chelator CQ (50 mg/kg/48 h). Six days after the zinc injection, the rats were sacrificed and the hippocampus was homogenized for Western blotting. Lower, quantitative analysis (n = 4). **, *p*<0.01 vs. control group; #, *p*<0.05 vs. zinc injected group.

To further confirm that zinc deficiency is involved in hippocampal BDNF deficit, we injected zinc sulfate into the rat brains, with or without intraperitoneal injection of zinc chelator CQ. The results showed that zinc treatment increased BDNF level in rat hippocampus, while simultaneous injection of CQ arrested the increase; furthermore, CQ alone also reduced the BDNF level (Fig. 5). These results indicate that certain level of zinc is required for BDNF expression, and zinc deficiency decreases BDNF level.

## Discussion

At a time when dietary fortification and supplementation of zinc have increased the zinc consuming level especially in the youth, the possible toxicity of zinc overdose on brain function remained largely unknown. In the present study, we explored for the first time the brain zinc homeostasis in zinc-enhanced animals in details, and further disclosed the mechanisms underlying high dose zinc intake-induced memory impairment.

We used weaning ICR mice as research models, since compared with inbred mice, mice in closed group showed bigger genetic variations, which may mimic the human zinc supplement users in a better way. The dose of low zinc supplementation (15 ppm) was chosed according to the recommended supplementation dosage in human (15 mg/d), high dose zinc intake was designed to be four times of the low dosage (60 ppm). Previous reports have showed that there was no serious toxicity of chronic oral zinc intake in an extremely high dosage (ZnSO_4_·7H_2_O 20 mg/ml, which equals to about 4530 ppm Zn in drink water) in C57BL/6 mice for 27 days [30] and in another transgenic mice for 6 months [31], except for body weight loss and fur color changes. In our experiment, we also did not observe any overt signs of serious toxicity, the body weight showed no change in high dose zinc enhancement group (data not shown), so our choice for the high dose of zinc supplementation is reasonable.

Consistent with previous reports, zinc supplementation in low dosage may increase the anxiety level [19], [32].The previous data regarding the low dose zinc supplementation on learning and memory was conflicting. Flinn JM *et al.* reported in a series of publications that enhanced zinc (10 ppm) consumption causes memory deficits in rats [19], [32] and potentiates memory impairment in transgenic disease mouse models [33], [34], while others observed improved performance of the animals in spatial memory tasks [35], [36]. In our experiments, we also observed improved performance of mice in contextual discrimination task. The underlying mechanism for the memory improvement by low dose zinc supplement needs further exploration. On the contrary, zinc supplementation in high dose resulted in impaired spatial memory. Interestingly, the memory deficit seemed to be highly hippocampus dependent, since high dose supplementation of zinc only impaired the performance of the mice in context discrimination but not in contextual conditioning. In a carefully designed experiment by Frankland *et al.* from Cold Spring Harbor Laboratory, contextual discrimination was shown to be a more sensitive measure of hippocampus dysfunction compared with the classical contextual conditioning, the latter, can be mediated by both hippocampus and nonhippocampus neural systems [29].So the results here indicate that the hippocampus, the brain region containing the highest level of zinc [37], is sensitive to oral zinc enhancement.

To further elucidate the mechanisms underlying the hippocampus dysfunction induced by zinc enhancement, we explored the changes of zinc homeostasis in the brain. Mice fed high dietary zinc showed significantly increased zinc level in serum and hemocytes, but the total brain zinc level was not changed. This result is consistent with previous research in which BL6/DBA mice were fed high zinc diets (300–1000 ppm) for 7–17 months, the brain zinc only showed a trend but no significant elevation [38]. Another study performed by Wang *et al.* showed a significant increase of brain zinc in transgenic APP/PS1 mice fed a high zinc diet [31], but the dosage of supplemented zinc was extremely high (ZnSO4·7H2O 20 mg/ml in drink water, which equals to about 4530 ppm Zn), which may not occur in human. And we suspected such a high dose zinc intake had exceeded the compensatory ability of the brain to maintain the zinc homeostasis.

It seemed that the total brain zinc was controlled strictly by some regulatory effects, and it may not be easily influenced by peripheral zinc level. In supporting this idea, severe dietary zinc deficiency also only induced decrease of serum zinc but not total brain zinc level [39], [40]. In one of these dietary zinc deprivation experiments, the author found no change of total brain zinc but significantly reduced hippocampal zinc level [40], indicating that hippocampus is sensitive to peripheral zinc fluctuation. So we further detected the hippocampal zinc levels. The result showed that the hippocampal zinc level detected by atomic absorption spectrophotometry was dramatically decreased in high dose zinc intake mice. Zinc imaging by two methods which detect the chelatable zinc ions, Timm staining [41] and TSQ staining, further confirmed that synaptic zinc was significantly decreased, especially in the CA3 and dentate gyrus (DG) in hippocampus. This result is somewhat unexpected to our speculation, but may partly explain the contextual discrimination impairment of the animals, because the DG-to-CA3 pathway play a key role in pattern separation both in rodent and human [42], and synaptic zinc release in this pathway is essential for LTP induction and hippocampus-dependent spatial learning and memory [12], [13], [14], [15]. Genetic depletion of synaptic zinc in mice by knocking out the synapse-specific vesicular zinc transporter ZnT3 (ZnT3KO mice) also induced complete deficits in contextual discrimination and spatial working memory [25]. Thus, the synaptic zinc deficiency may underlie the hippocampus-dependent memory impairment in high level zinc enhanced animals. The upstream mechanisms for hippocampal zinc deficiency need to be further investigated. One possible reason is that the zinc-containing hippocampal neurons are vulnerable to zinc toxicity; we have observed decreased cell numbers in hippocampal CA3 area by Nissl staining in the high dose group (data not shown). Another speculation is that the zinc uptake system (especially zinc inward transporters) in the hippocampus is specifically impaired by peripheral zinc overdose.

The formation and consolidation of memory require the participation of some central molecular devices such as glutamate receptors and synaptic scaffolding proteins [43], [44].Previous study had shown that zinc deficiency might reduce NMDA receptor expression in rat brains [39], Adlard *et al.* also observed significantly decreased AMPA, NMDA-NR2A and NR2B receptors and scaffolding PSD-95 levels in old ZnT3KO mice [45]. Considering that zinc overdose induced hippocampal zinc deficiency, we suspect that these memory-related proteins are also disturbed. Consistent with our hypothesis, the protein levels of AMPA-GluR1, NMDA-NR2A, NR2B, PSD-93 and PSD-95 were all obviously reduced in hippocampus, AMPA-GluR2 also showed an un-significant decrease. At the same time, significant loss of dendritic spines was observed. All these proteins play an important role in the formation of new spines and in learning/memory, so besides the deficiency of synaptic zinc release, disturbed expression of learning and memory related receptors and scaffolding proteins also contributed to the spatial memory impairment in high level zinc intake animals.

We further explored mechanisms for the compromised expression of learning and memory related proteins, and found that BDNF-TrkB neurotrophic signaling was specifically impaired in the hippocampus of high dose zinc supplemented mice. BDNF was reported to regulate the expression and traffic of NMDA receptors and synaptic scaffolding proteins [46], [47], [48], and a large body of evidences from human and BDNF knockout mice supported that BDNF plays a key role in hippocampal function and hippocampus-dependent memory [49], [50], [51]. Previous studies have showed that zinc may induce BDNF expression [52], [53], and activity-dependent release of extracellular zinc could also potentiate the BDNF-TrkB signaling pathway by activation of metalloproteinases, which convert pro-BDNF to mature BDNF [54]. Furthermore, the decreased expression of NMDA receptors and scaffolding proteins both in dietary zinc deficiency and genetic synaptic zinc depletion mice was accompanied with reduced BDNF level [39], [45]. All these studies, together with our data, strongly suggested that high dose zinc supplementation-induced hippocampal zinc deficiency may impair the memory through BDNF deficit. To further confirm this hypothesis, we injected zinc into the rat brain directly, with or without administration of zinc chelator CQ. The result showed that zinc mediated the increase of BDNF expression, while zinc chelating reduced the BDNF level in hippocampus. Taking together, zinc overdose results in hippocampal synaptic zinc deficiency, which further reduce BDNF-TrkB neurotrophic signaling, the latter, promoted the memory impairment.

**Figure 6:**
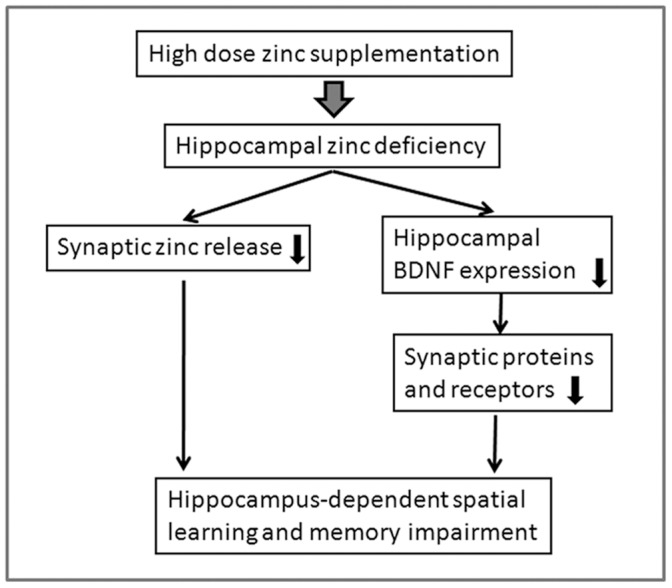
Schematic illustration showing the mechanisms underlie the high dose zinc supplementation-induced memory deficits. Hippocampus is susceptible to zinc enhancement. High dose supplementation of zinc induces specific zinc deficiency in hippocampus, which impair learning and memory due to decreased release of synaptic zinc. Hippocampal zinc deficiency also induces BDNF signaling deficit, which resulted in decreased expression of synaptic proteins and receptors, the latter, further contributes to learning and memory impairment.

In a summary, synaptic zinc plays an important role in hippocampus-dependent learning and memory and BDNF expression, and hippocampus is susceptible to zinc enhancement. High dose supplementation of zinc induces specific zinc deficiency in hippocampus, which further impair learning and memory due to decreased availability of synaptic zinc and BDNF deficit (Fig. 6). Thus, zinc supplement users should take caution to avoid the overuse of zinc supplementation.
